# Identification of the methotrexate resistance-related diagnostic markers in osteosarcoma via adaptive total variation netNMF and multi-omics datasets

**DOI:** 10.3389/fgene.2023.1288073

**Published:** 2023-10-23

**Authors:** Zhihan Jiang, Kun Han, Daliu Min, Wei Kong, Shuaiqun Wang, Min Gao

**Affiliations:** ^1^ College of Information Engineering, Shanghai Maritime University, Shanghai, China; ^2^ Department of Medical Oncology, The Sixth People’s Hospital Affiliated to Shanghai Jiaotong University School of Medicine, Shanghai, China

**Keywords:** osteosarcoma, non-negative matrix factorization, adaptive total variation, methotrexate resistance, diagnostic markers

## Abstract

Osteosarcoma is one of the most common malignant bone tumors with high chemoresistance and poor prognosis, exhibiting abnormal gene regulation and epigenetic events. Methotrexate (MTX) is often used as a primary agent in neoadjuvant chemotherapy for osteosarcoma; However, the high dosage of methotrexate and strong drug resistance limit its therapeutic efficacy and application prospects. Studies have shown that abnormal expression and dysfunction of some coding or non-coding RNAs (e.g., DNA methylation and microRNA) affect key features of osteosarcoma progression, such as proliferation, migration, invasion, and drug resistance. Comprehensive multi-omics analysis is critical to understand its chemoresistant and pathogenic mechanisms. Currently, the network analysis-based non-negative matrix factorization (netNMF) method is widely used for multi-omics data fusion analysis. However, the effects of data noise and inflexible settings of regularization parameters affect its performance, while integrating and processing different types of genetic data is also a challenge. In this study, we introduced a novel adaptive total variation netNMF (ATV-netNMF) method to identify feature modules and characteristic genes by integrating methylation and gene expression data, which can adaptively choose an anisotropic smoothing scheme to denoise or preserve feature details based on the gradient information of the data by introducing an adaptive total variation constraint in netNMF. By comparing with other similar methods, the results showed that the proposed method could extract multi-omics fusion features more effectively. Furthermore, by combining the mRNA and miRNA data of methotrexate (MTX) resistance with the extracted feature genes, four genes, Carboxypeptidase E (*CPE*), LIM, SH3 protein 1 (*LASP1*), Pyruvate Dehydrogenase Kinase 1 (*PDK1*) and Serine beta-lactamase-like protein (*LACTB*) were finally identified. The results showed that the gene signature could reliably predict the prognostic status and immune status of osteosarcoma patients.

## 1 Introduction

Osteosarcoma is one of the most common malignant bone cancers, accounting for approximately 30% of all osteosarcomas and mainly affecting children and adolescents, with a peak incidence at age 18 (Sadykova et al., 2020). Neoadjuvant chemotherapy (NAC) consisting of methotrexate, doxorubicin (also known as adriamycin), and cisplatin is referred to as MAP (Benjamin, 2020). The combination of NAC and surgical resection has significantly increased the 5-year survival rate for patients with osteosarcoma from 20% to 70% (Chen et al., 2021). However, up to 20% of patients develop resistance to this treatment regimen (Bacci et al., 2000), and their 5-year survival rate is extremely poor, at around 20% (Prudowsky and Yustein, 2020). Therefore, comprehensive analysis of multi-omics genetic data of osteosarcoma, screening for differentially expressed genes (DEGs) associated with drug resistance and analysis of the impact of DEGs on prognosis are essential for finding new targets to improve overall survival and reverse drug resistance.

Malignant osteosarcoma cells are strongly associated with chemoresistance, recurrence, and metastatic processes (Schiavone et al., 2019; Mutsaers and Walkley, 2014), and osteosarcomas have significant heterogeneity at the genomic, transcriptomic, and epigenetic levels resulting from abnormal epigenetic modifications (Sun et al., 2023). For example, methylation levels and miRNA dysfunction have been identified as characteristic events in human osteosarcoma cell lines, with higher methylation events associated with more severe phenotypes (de Azevedo et al., 2019). Abnormal DNA methylation can affect gene expression, cell cycle, and apoptosis and regulate the development and progression of osteosarcoma by inhibiting transcription (Wang et al., 2020). miRNAs (microRNAs) are endogenous small non-coding RNAs that play critical regulatory roles in various biological processes, including differentiation, cell proliferation, cell cycle control, apoptosis, drug resistance, and innate immunity (Mens and Ghanbari, 2018; Patil et al., 2013). Although many studies have identified DNA methylation in osteosarcoma as an important therapeutic target, the reasons why DNA methylation, miRNAs, and target genes combine to lead to chemoresistance and poorer prognosis remain to be determined.

Currently, non-negative matrix factorization (NMF) and its various improvements are widely used in a single type of genetic data analysis. For example, Lei et al. applied NMF to osteosarcoma gene data analysis and identified molecular subgroups with different Ferroptosis-related gene expression patterns (Lei et al., 2021). Jiao et al. proposed a hypergraph regularization constraint-based NMF method (HC-NMF) to select differentially expressed genes and classify tumor samples (Jiao et al., 2020). Leng et al. proposed an adaptive total-variance constraint-based NMF method (ATV-NMF), which can adaptively denoise or maintain feature details based on gradient information (Leng et al., 2017). Zhu et al. applied ATV-NMF to single-cell sequencing data clustering and achieved accurate results in cell subpopulation clustering and the identification of marker genes (Zhu et al., 2021). However, these improved NMF-based methods do not consider the relationship between different types of genetic data and cannot integrate and decompose different types of genetic data simultaneously. To address this issue, Zhang et al. used a joint NMF (jNMF) approach to integrate DNA methylation (ME), GE, and miRNA expression data from ovarian cancer to identify ovarian cancer-related multi-dimensional modules (Zhang et al., 2012). Liu et al. proposed a TriNMF-based network-assisted co-clustering method for cancer subtype identification (NCIS) that incorporates molecular interaction networks into the clustering process to improve the identification of cancer subtypes (Liu et al., 2014; Ding et al., 2006). Chen proposed the netNMF method based on NMF using a network framework to identify co-expression modules of two different types of genetic data (Chen and Zhang, 2018). NetNMF uses the decomposed submatrices to construct co-expression networks, which may weaken the connectivity of the nodes in the network. Therefore, Zhuang et al. proposed a hypergraph regularization constraint-based netNMF method (HG-netNMF) (Zhuang et al., 2023), and Ding et al. proposed a graph regularization-based netNMF method (NMFNA), both of which can better mine higher-order features between two genetic data compared to netNMF (Ding et al., 2021). The above NMF-based network analysis method provides an effective way to understand the interactions of different genetic data to understand the pathogenic mechanisms of cancer.

In this study, we proposed an improved NMF network analysis method (ATV-netNMF) to integrate DNA methylation and gene expression data. On this basis, combined with the miRNA and mRNA data of MTX resistance, we built a signature of MTXDEGs that predicted the prognosis of osteosarcoma, and the results revealed that the high-risk group had fewer immune cells and a lower degree of immune infiltration, which could lead to a poor prognosis.

## 2 Materials and methods

### 2.1 Workflow of this study

**FIGURE 1:**
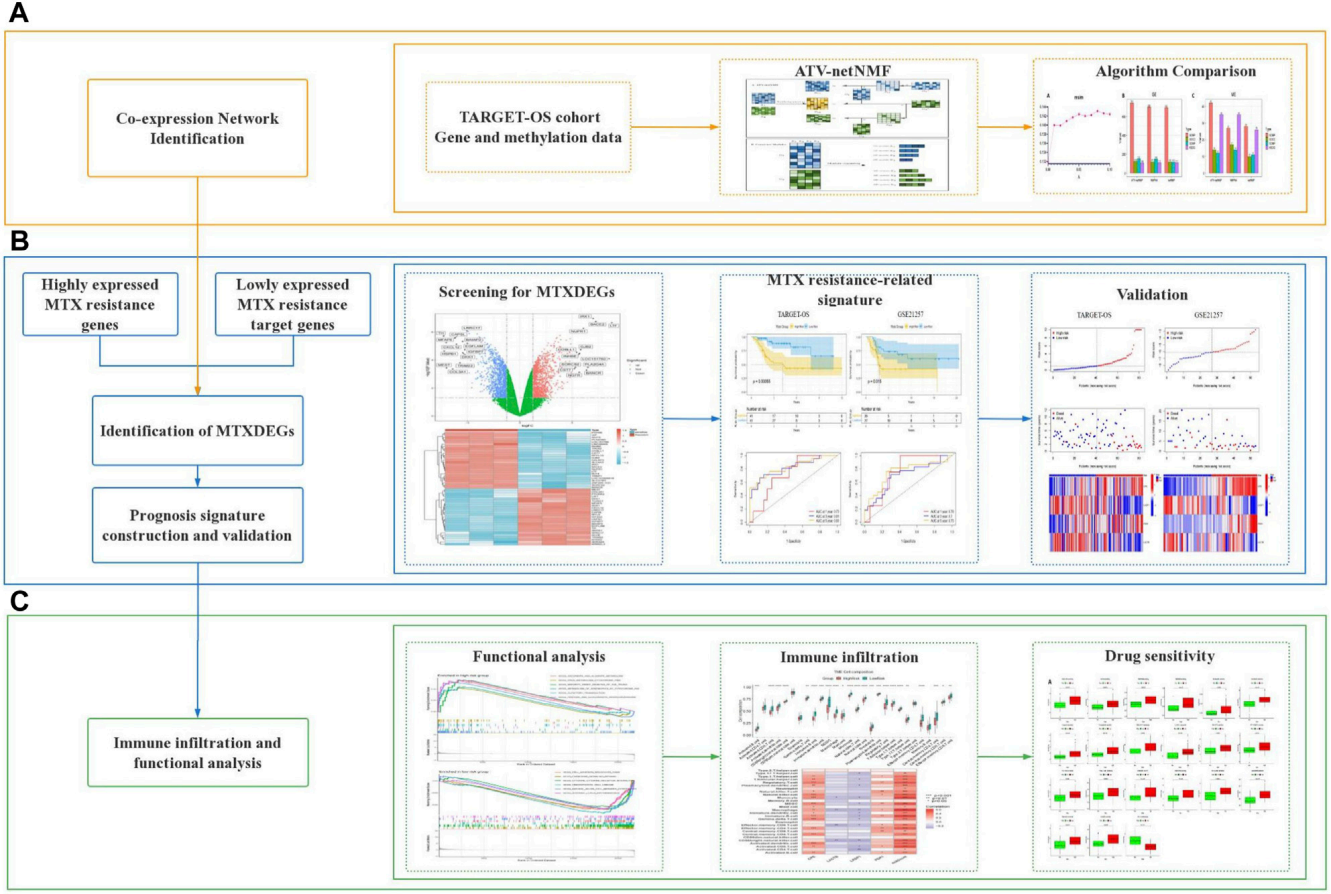
Workflow of this study. **(A)** Co-expression network analysis. **(B)** Identification of MTXDEGs and gene signature construction. **(C)** Functional analysis, immune infiltration analysis and drug sensitivity analysis.

This study is mainly divided into three stages, in the first stage, to efficiently fuse methylation and gene expression data, the proposed ATV-netNMF was applied to two types of genetic data to identify co-expression networks and core gene modules that are strongly associated with variation in both data. Furthermore, the core module was analyzed by KEGG and GO enrichment to compare with other methods based on the number of pathways enriched and pathway significance (Figure 1A). In the second stage, considering the degradation and inhibitory effects of miRNAs, target genes were predicted using upregulated miRNAs intersecting with downregulated genes and core module genes taken as target genes regulated by MTX-resistant miRNAs. Genes highly expressed in MTX-resistant osteosarcoma cells were obtained using the intersection of upregulated genes and core module genes. Finally, the two parts of genes were considered together as MTXDEGs. Then, the extracted MTXDEGs were used to construct the gene signature and calculate the risk scores, which were validated for their predictive performance using an independent dataset (Figure 1B). In the third stage, the risk scores were used to classify the samples into high-risk and low-risk groups for functional analysis, immune infiltration analysis, and drug sensitivity analysis (Figure 1C).

### 2.2 Data sources

82 osteosarcoma patients with complete clinical characteristics, methylation data, and gene expression data were obtained from the TARGET database (https://portal.gdc.cancer.gov/) as the training cohort, and 53 osteosarcoma patients with RNA-seq and clinical characteristics from GSE21257 in the GEO database (https://www.ncbi.nlm.nih.gov/geo) as the validation cohort. GSE16089 (Selga et al., 2009) and GSE223857 (Zhang et al., 2023a) included three methotrexate-resistant and three methotrexate-sensitive osteosarcomas mRNA and miRNA data in the GEO database.

### 2.3 Adaptive total-variation constrained based netNMF

The typical NMF (Lee and Seung, 1999) decomposes the nonnegative matrix $V^{m\times n}$ into two nonnegative matrices $W^{m\times k}$ and $H^{k\times n}$ , where $W^{m\times k}$ is the basis matrix and $H^{k\times n}$ is the loading matrix, such that V≈WH , where $k<\min\left(m,n\right)$ . To minimize the factorization error between ***V*** and ***WH***, which can be written as,

$$\begin{array}{l} \min\\ W,H \end{array}{\left\|V-WH\right\|}_{F}^{2}$$ (1)

s.t. W≥0,H≥0

On the basis of two and three-factor NMF( V≈FSG ) (Ding et al., 2006), if ***V*** is a symmetric similarity matrix, it could be decomposed into $GSG^{T}$ . For biological networks with the same samples but with two different types of features, combining the above ideas, netNMF (Chen and Zhang, 2018) is defined as,

$$\begin{array}{l} \min\\ G_{1},G_{2},S_{11},S_{22} \end{array}{\left\|R_{11}-G_{1}S_{11}G_{1}^{T}\right\|}_{F}^{2}+\lambda_{1}{\left\|R_{12}-G_{1}G_{2}^{T}\right\|}_{F}^{2}+\lambda_{2}{\left\|R_{22}-G_{2}S_{22}G_{2}^{T}\right\|}_{F}^{2}$$

$$s.t.\;G_{1}\geq 0,G_{2}\geq 0,S_{11}\geq 0,S_{22}\geq 0$$ (2)

NMFNA (Ding et al., 2021) applies graph regularization constraints in netNMF that can discover and enhance the inherent geometric data structure and improve the ability to identify modules. Based on netNMF and graph regularization constraints, the objective function of NMFNA is defined as,

$$\begin{array}{c} \begin{array}{l} \min\\ G_{1},G_{2},S_{11},S_{22} \end{array}{\left\|R_{11}-G_{1}S_{11}G_{1}^{T}\right\|}_{F}^{2}+\alpha {\left\|R_{12}-G_{1}G_{2}^{T}\right\|}_{F}^{2}+\beta {\left\|R_{22}-G_{2}S_{22}G_{2}^{T}\right\|}_{F}^{2}\\ +\sum_{i=1}^{2}\lambda_{i}Tr\left(G_{i}^{T}L_{i}G_{i}\right)s.t.\;G_{1}\geq 0,G_{2}\geq 0,S_{11}\geq 0,S_{22}\geq 0 \end{array}$$ (3)

where $R_{11}^{n1\times n1}$ and $R_{22}^{n2\times n2}$ are the autocorrelation matrices of $X_{1}\text{ and }X_{2}$ which are symmetric similarity matrices corresponding to the two features, and $R_{12}^{n1\times n2}$ is the intercorrelation matrix between them, which are all non-negative; $G_{1}^{n1\times k}$ and $G_{1}^{n2\times k}$ are non-negative matrices identifying the feature modules in their respective networks. $S_{11}^{k\times k}$ and $S_{22}^{k\times k}$ are symmetric non-negative decomposition matrices; *k* is a prespecified dimensionality reduction parameter; ***L*** is the graph Laplacian matrix; λ is used to adjust the strength of the graph regularization constraint; α and β are used to balance the first three terms of the objective function, which are set to $n_{1}/n_{2}$ and ${\left(n_{1}/n_{2}\right)}^{2}$ by default.

In order to better remove data noise and retain key feature details, we propose the ATV-netNMF method, which can improve the tolerance of the algorithm to noise and improve the performance of the algorithm by introducing adaptive total-variation constraint on NMFNA. Adaptive total variation (Leng et al., 2017; Levine, 2005) can be adapted based on gradient information for denoising or preserving feature details, which can be illustrated as,

$$E\left(G\right)={\left\|G\right\|}_{ATV}=\int_{\Omega }\frac{1}{p\left(x,y\right)}{\left|\nabla G\right|}^{p\left(x,y\right)}dxdy$$ (4)

$$p\left(x,y\right)=1+{\left(1+{\left|\nabla G\right|}^{2}\right)}^{-1},1<p\left(x,y\right)<2$$ (5)

where E is the energy function of ***G***, ${\left\|G\right\|}_{ATV}=\int_{\Omega }\frac{1}{p\left(x,y\right)}{\left|\nabla G\right|}^{p\left(x,y\right)}dxdy$ represents the adaptive total-variation regularization term, and $\left(\nabla G\right)\left(i,j\right)=\left(\left(\partial_{x}G\right)\left(i,j\right),\left(\partial_{y}G\right)\left(i,j\right)\right)$ is the discrete gradient form with $\left(\left(\partial_{x}G\right)\left(i,j\right),\left(\partial_{y}G\right)\left(i,j\right)\right)$ , is given by

$$\left(\partial_{x}G\right)\left(i,j\right)=\left\{\begin{array}{l} G\left(i+1,j\right)-G\left(i,j\right)\text{ if }i<r\\ G\left(1,j\right)-G\left(r,j\right)\text{ if }i=r \end{array}\right.$$ (6)

$$\left(\partial_{y}G\right)\left(i,j\right)=\left\{\begin{array}{l} G\left(i,j=1\right)-G\left(i,j\right)\text{ if }j<n\\ G\left(i,1\right)-G\left(i,n\right)\text{ if }j=n \end{array}\right.$$ (7)

Based on the NMFNA and the adaptive total variation constraint, the objective function of the ATV-netNMF is defined as,

$$\begin{array}{c} \begin{array}{l} \min\\ G_{1},G_{2},S_{11},S_{22} \end{array}{\left\|R_{11}-G_{1}S_{11}G_{1}^{T}\right\|}_{F}^{2}+\alpha {\left\|R_{12}-G_{1}G_{2}^{T}\right\|}_{F}^{2}+\beta {\left\|R_{22}-G_{2}S_{22}G_{2}^{T}\right\|}_{F}^{2}\\ +\sum_{i=1}^{2}\left[\lambda_{i}Tr\left(G_{i}^{T}L_{i}G_{i}\right)+2{\left\|G_{i}\right\|}_{ATV}\right]s.t.\;G_{1}\geq 0,G_{2}\geq 0,S_{11}\geq 0,S_{22}\geq 0 \end{array}$$ (8)

Where $R_{11}\text{ and }R_{22}$ are ME and GE co-expression networks, $R_{12}$ is ME-GE co-expression network, other symbolic meanings and parameter settings are the same as NMFNA.

This study uses the multiplicative iterative update algorithm to minimize the objective function of ATV-netNMF. Suppose $B_{1},B_{2},B_{3}\text{ and }B_{4}$ are matrices of Lagrange multipliers which constrain $S_{11}\geq 0,S_{22}\geq 0,G_{1}\geq 0\text{ and }G_{2}\geq 0$ respectively, and the Lagrangian function f of ATV-netNMF is

$$\begin{array}{l} f=tr\left({\left(R_{11}-G_{1}S_{11}G_{1}^{T}\right)}^{T}\left(R_{11}-G_{1}S_{11}G_{1}^{T}\right)\right)+\alpha tr\left({\left(R_{12}-G_{1}G_{2}^{T}\right)}^{T}\left(R_{12}-G_{1}G_{2}^{T}\right)\right)\\ +\beta tr\left({\left(R_{22}-G_{2}S_{22}G_{2}^{T}\right)}^{T}\left(R_{22}-G_{2}S_{22}G_{2}^{T}\right)\right)+\lambda_{1}Tr\left(G_{1}^{T}L_{1}G_{1}\right)+\lambda_{2}Tr\left(G_{2}^{T}L_{2}G_{2}\right)\\ +2{\left\|G_{1}\right\|}_{ATV}+2{\left\|G_{2}\right\|}_{ATV}+tr\left(B_{1}^{T}S_{11}\right)+tr\left(B_{2}^{T}S_{22}\right)+tr\left(B_{3}^{T}G_{1}\right)+tr\left(B_{4}^{T}G_{2}\right) \end{array}$$ (9)

Thus, the partial derivatives of f with respect to $S_{11},S_{22},G_{1},\text{and }G_{2}$ are,

$$\frac{\partial f}{\partial S_{11}}=-2G_{1}^{T}R_{11}G_{1}+2G_{1}^{T}G_{1}S_{11}G_{1}^{T}G_{1}+B_{1}$$ (10)

$$\frac{\partial f}{\partial S_{22}}=-2G_{2}^{T}R_{22}G_{2}+2G_{2}^{T}G_{2}S_{22}G_{2}^{T}G_{2}+B_{2}$$ (11)

$$\frac{\partial f}{\partial G_{1}}=4\left(G_{1}S_{11}G_{1}^{T}G_{1}S_{11}-R_{11}G_{1}S_{11}\right)+2\alpha \left(G_{1}G_{2}^{T}G_{2}-R_{12}G_{2}\right)+2\lambda_{1}L_{1}G_{1}-2\mathrm{div}\left(\frac{\nabla G_{1}}{{\left|\nabla G_{1}\right|}^{2-p}}\right)+B_{3}$$ (12)

$$\frac{\partial f}{\partial G_{2}}=4\beta \left(G_{2}S_{22}G_{2}^{T}G_{2}S_{22}-R_{22}G_{2}S_{22}\right)+2\alpha \left(G_{2}G_{1}^{T}G_{1}-R_{12}G_{1}\right)+2\lambda_{2}L_{2}G_{2}-2\mathrm{div}\left(\frac{\nabla G_{2}}{{\left|\nabla G_{2}\right|}^{2-p}}\right)+B_{4}$$ (13)

Let $B_{1}S_{11}=0,B_{2}S_{22}=0,B_{3}G_{1}=0,B_{4}G_{2}=0$ , the iterative formula can be written as,

$${\left(S_{11}\right)}_{kk}\leftarrow {\left(S_{11}\right)}_{kk}\frac{{\left(G_{1}^{T}R_{11}G_{1}\right)}_{kk}}{{\left(G_{1}^{T}G_{1}S_{11}G_{1}^{T}G_{1}\right)}_{kk}}$$ (14)

$${\left(S_{22}\right)}_{kk}\leftarrow {\left(S_{22}\right)}_{kk}\frac{{\left(G_{2}^{T}R_{22}G_{2}\right)}_{kk}}{{\left(G_{2}^{T}G_{2}S_{22}G_{2}^{T}G_{2}\right)}_{kk}}$$ (15)

$${\left(g_{1}\right)}_{ik}\leftarrow {\left(g_{1}\right)}_{ik}\frac{{\left(\alpha R_{12}G_{2}+2R_{11}G_{1}S_{11}+\mathrm{div}\left(\frac{\nabla G_{1}}{{\left|\nabla G_{1}\right|}^{2-p}}\right)\right)}_{ik}}{{\left(2G_{1}S_{11}G_{1}^{T}G_{1}S_{11}+\alpha G_{1}G_{2}^{T}G_{2}+2\lambda_{1}L_{1}G_{1}\right)}_{ik}}$$ (16)

$${\left(g_{2}\right)}_{kj}\leftarrow {\left(g_{2}\right)}_{kj}\frac{{\left(\alpha R_{12}G_{1}+2\beta R_{22}G_{2}S_{22}+\mathrm{div}\left(\frac{\nabla G_{2}}{{\left|\nabla G_{2}\right|}^{2-p}}\right)\right)}_{kj}}{{\left(2\beta G_{2}S_{22}G_{2}^{T}G_{2}S_{22}+\alpha G_{2}G_{1}^{T}G_{1}+2\lambda_{2}L_{2}G_{2}\right)}_{kj}}$$ (17)

**FIGURE 2:**
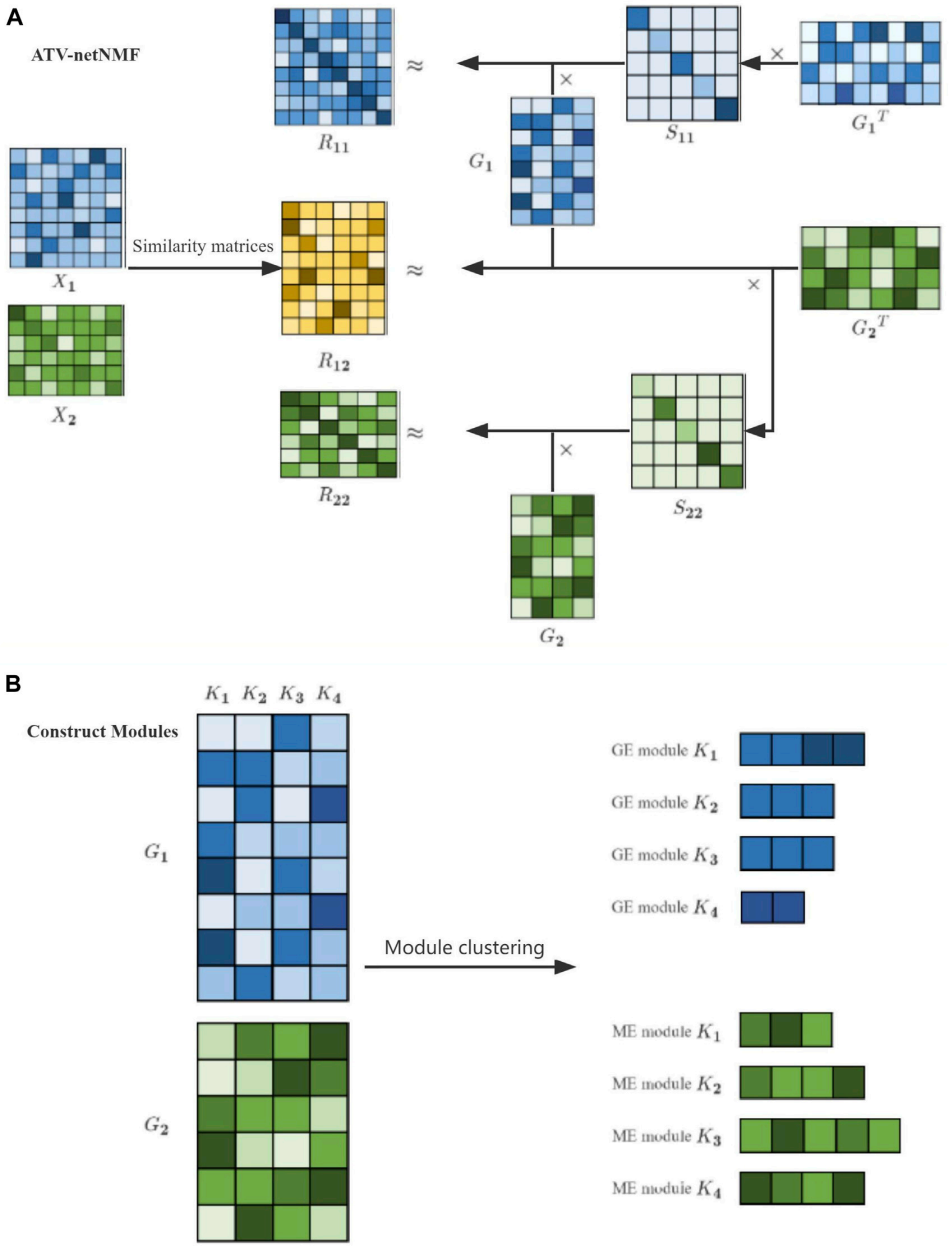
Workflow of ATV-netNMF. **(A)** ATV-netNMF decomposes multi-omics data. **(B)** Identification of co-expression modules.

Where div denotes the divergence of the matrix, ∇G denotes the gradient of the matrix, and $\left|\nabla G\right|$ denotes the norm of the gradient of the matrix. The adaptive total variation regularization includes a diffusion coefficient $\frac{1}{{\left|\nabla G\right|}^{2-p}}$ in Eqs 18, 19, which controls the data diffusion rate based on gradient information. For data edges, larger values of ${\left|\nabla G\right|}^{2-p}$ and smaller values of $\frac{1}{{\left|\nabla G\right|}^{2-p}}$ help to maintain the edges. In data smoothing regions, smaller values of ${\left|\nabla G\right|}^{2-p}$ and larger values of $\frac{1}{{\left|\nabla G\right|}^{2-p}}$ help to remove noise (Leng et al., 2017). ATV can preserve or enhance data features while removing noise. The overall workflow of ATV-netNMF is shown in (Figure 2A). Firstly, three co-expression networks are constructed using ME and GE data. Then the network is decomposed using the objective function to identify the co-expression modules under the guidance of $G_{1}$ and $G_{2}$ (Figure 2B), z-scores of each column vector of $g_{i}$ are calculated as follows.

$$g^{*}=\frac{g-\bar{g}}{\frac{1}{n-1}\sum_{i}{\left(g_{i}-\bar{g}\right)}^{2}}$$ (18)

$$\bar{g}=\frac{1}{n}\sum_{i}g_{i}$$ (19)

The genes with each column weight greater than or equal to the threshold are used as module members, the threshold is set to 2, and the module with the most genes called the core module according to the previous study (Ding et al., 2021).

### 2.4 Analysis of differentially expressed genes (DEGs) and miRNAs (DE-miRNAs)

The R package “limma” (Chen et al., 2022) was used to find differentially expressed genes and miRNAs between methotrexate-resistant and sensitive cells in the GSE16089 and GSE223857 datasets, with the threshold set to |Log2FC|>1 and *p* < 0.05.

### 2.5 Prediction of miRNA targeted genes

The target genes of upregulated miRNAs were predicted by TargetScan (http://www.targetscan.org/) and miRDB (http://mirdb.org/miRDB/) online databases, and the screening threshold was set as the cut-off parameter for TargetScan was the total contex++ score < - 0.05, while miRdb score >50.

### 2.6 Construction and validation of a MTX resistance-related signature in osteosarcoma

Statistically, significant (*p* < 0.05) MTXDEGs associated with OS prognosis were obtained by univariate COX regression analysis using the survival package in R. The least absolute shrinkage and selection operator (LASSO) regression analysis was performed on prognosis-related MTXDEGs using the glmnet package in R to reduce the dimensionality of genes in the model. Subsequently, independent prognostic genes were screened using multivariate Cox regression analysis, and regression coefficients for the corresponding genes were generated. A linear combination of gene expression levels and regression coefficients created a signature with the following formula for the risk score.

$$Riskscore=\sum_{i=1}^{n}\mathrm{Exp}\left(i\right)*coef\left(i\right)$$ (20)

The median of Riskscore was used to determine the best critical value to classify patients into high-risk and low-risk groups. Kaplan-Meier survival curves and time-dependent receptor operating characteristic (ROC) curves were used to assess the predictive performance of prognostic signatures on overall survival. GSE21257 was used as a validation set to verify the predictive performance of the signature.

### 2.7 Construction of the nomogram based on prognostic models

Compared with other clinical characteristics (including metastasis, race, age, and gender), univariate and multivariate COX analyses were performed to determine the independence of our established gene signature in predicting overall survival, and *p* < 0.05 was considered statistically significant. To predict the prognosis of patients with osteosarcoma, a nomogram integrating riskscore and clinical characteristics was constructed, and calibration curves were used to evaluate the predictive accuracy of the nomogram, which was constructed from the “rms” R package (Liu et al., 2023a). To estimate the clinical robustness of the MTX resistance gene signature, decision curve analysis (DCA) was used to calculate the net benefit of the signature for different threshold probabilities in the training and validation datasets.

### 2.8 Functional analysis

The R package limma was used to obtain differential genes between high-risk and low-risk groups, which were analyzed for GO and KEGG pathway enrichment using David (the Database for Annotation, Visualization and Integrated Discovery, https://david.ncifcrf.gov). The threshold for significantly enriched pathways was set to *p*-value <0.05, and the top 20 most significant pathways were selected.

### 2.9 Gene set enrichment analysis

Gene set enrichment analysis (GSEA) was performed to identify pathways enriched in the high-risk and low-risk groups to explore the relationship between riskscore and biological function, with the threshold of *p* < 0.05.

### 2.10 Evaluation of immune cell infiltration

A single sample gene set enrichment analysis (ssGSEA) (Liu et al., 2023b) method was used to analyze the differences in 28 immune cell infiltrates between the high-risk and low-risk groups. Tumor microenvironment analysis was performed on the gene expression data of osteosarcoma using an R package estimate (Zhang et al., 2023b) to obtain the immune score, stromal score, and estimate score for each patient, and the difference in scores between the high-risk and low-risk groups was analyzed. Correlation between immune cells and immune scores was performed using the ggstatsplot R package.

### 2.11 Drug sensitivity analysis

The OncoPredict (Maeser et al., 2021) R package was used to predict *in vivo* drug response in cancer patients, including half-maximal inhibitory concentration (IC50) values for 189 drugs corresponding to cell lines and a normalized gene expression matrix for 809 tumor cell lines from the Genomics of Drug Sensitivity in Cancer (GDSC) database. IC50 values for the TARGET-OS cohort were predicted using the oncoPredict method with a significance threshold set at *p* < 0.001.

## 3 Results

### 3.1 Screening of core modules by ATV-netNMF

The methylation and gene data of the TARGET-OS cohort had the same 82 osteosarcoma samples but with different features. Methylated genes with expression mean values less than 0.25 were filtered out from ME data, 15,819 methylated genes were retained, and 23,683 mRNAs were filtered out in GE data. Three co-expression networks were created from these data: ME network $R_{11}^{15819\times 15819}$ , ME-GE network $R_{12}^{15819\times 23683}$ , and GE network $R_{22}^{23683\times 23683}$ . Based on the previous study (Zhuang et al., 2023), the value of dimensionality reduction *k* generally is at most one-tenth of the minimum number of samples or features of the network modules. Therefore, in this study, *k* is set to 8, and the number of iterations is set to 200. By running the λ from 0 to 0.1, the highest module similarity is selected and set to $\lambda_{1}$ and $\lambda_{2}$ , and the module similarity (Wang et al., 2018) is defined as follows.

$$msim=\sum_{x,y}\frac{\left|M_{x}\cap M_{y}\right|}{\min\left(\left|M_{x},M_{y}\right|\right)}$$ (21)

**FIGURE 3:**
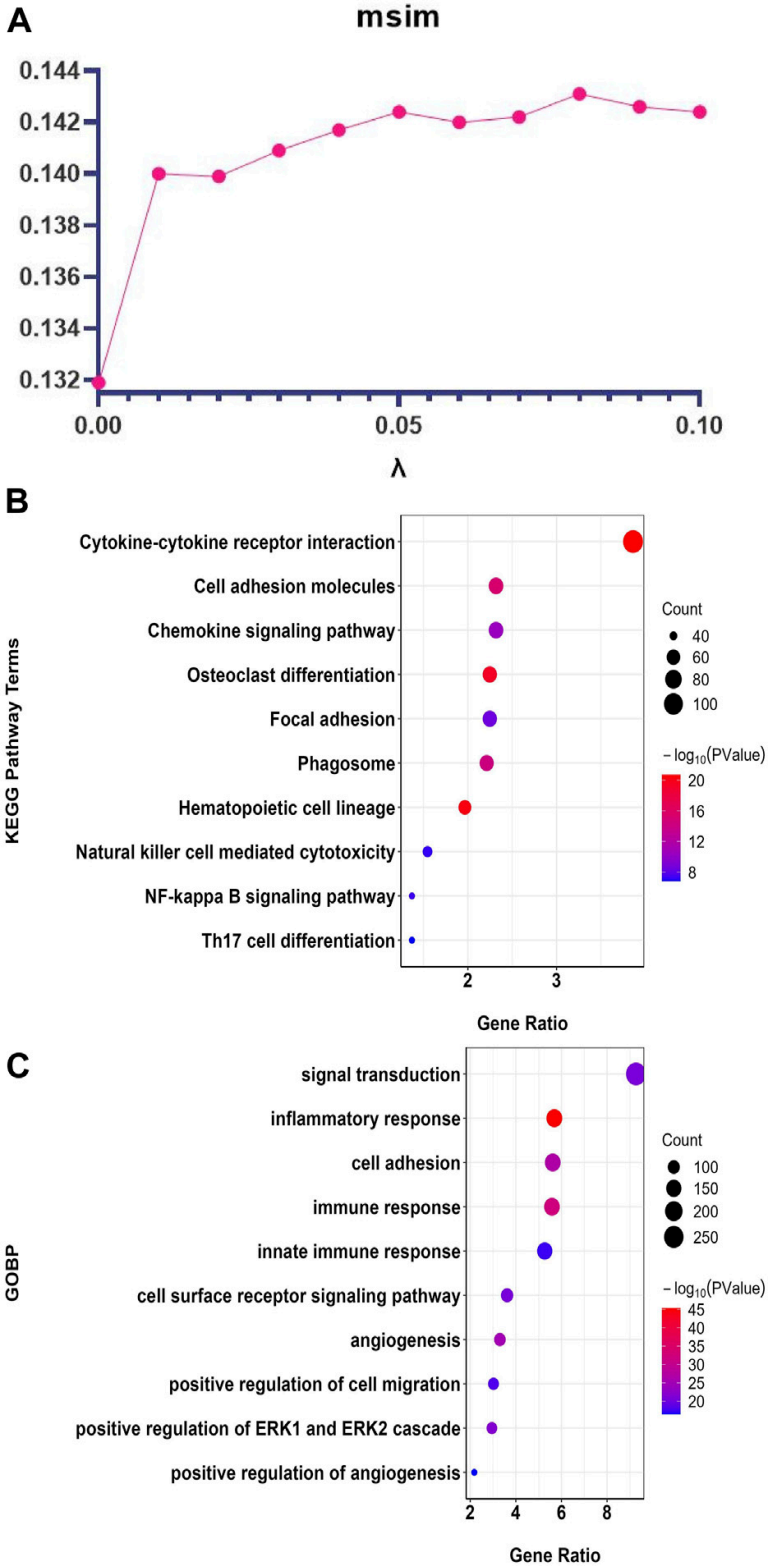
ATV-netNMF parameter setting and the top 10 enrichment results of the core GE modules identified by ATV-netNMF. **(A)** Setting of parameter λ . **(B)** Results of KEGG enrichment analysis. **(C)** Results of GOBP enrichment analysis.

$M_{x}$ denotes the members belonging to module x. According to Figure 3A, $\lambda_{1}、\lambda_{2}$ was set to 0.08. Finally, the core GE module containing 2810 mRNAs closely related to methylation data and the ME core module containing 1013 methylation genes were obtained by ATV-netNMF integrated analysis. The core gene module was selected for further analysis.

The top 10 enrichment terms of the core GE modules identified by ATV-netNMF are shown in Figure 3. The results showed that the genes were most enriched in terms related to tumor cell function and immune function. For example, KEGG was mainly enriched to cell adhesion molecules, osteoclast differentiation, NF-kappa B signaling pathway, Th17 cell differentiation (Figure 3B), and GOBP was mainly enriched to inflammatory response, immune response, cell adhesion, signal transduction (Figure 3C). The above results indicated that ATV-netNMF could effectively screen the gene network significantly related to the immune microenvironment of osteosarcoma.

### 3.2 Identification of MTXDEGs

**FIGURE 4:**
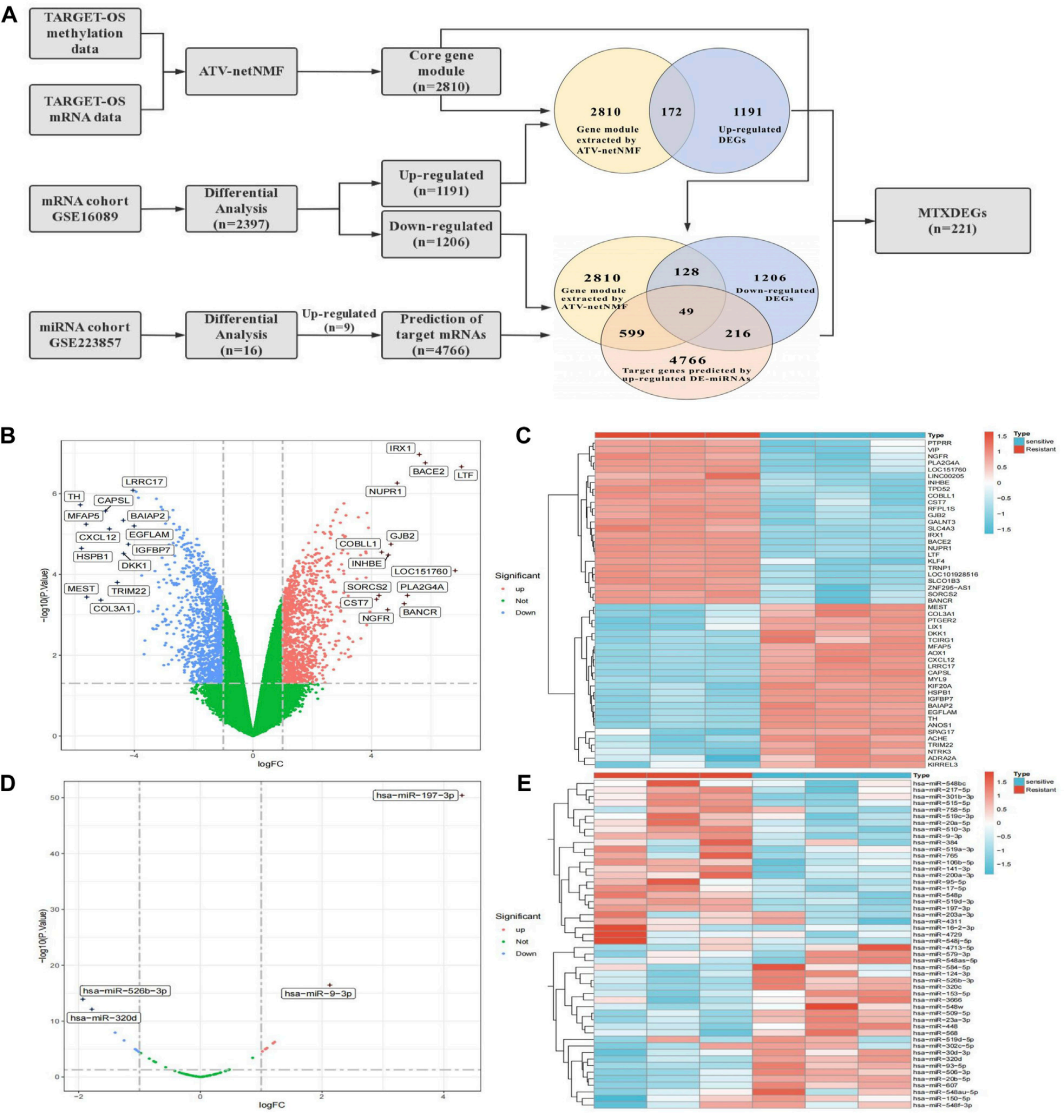
Identification of MTXDEGS. **(A)** Flowchart for identifying MTXDEGs. **(B,C)** Volcano and heat plot of DEGs. **(D,E)** Volcano and heat plot of DE-miRNAs.

The flowchart for the identification of MTXDEGs is shown in Figure 4A. We analyzed DEGs between methotrexate-resistant and sensitive osteosarcoma cells using GSE16089 and screened 2397 DEGs, of which 1191 were upregulated and 1206 were downregulated (Figures 4B, C). Then DE-miRNAs between methotrexate-resistant and sensitive osteosarcoma cells were analyzed using GSE223857, and 16 DE-miRNAs were screened, of which 9 were upregulated and 7 were downregulated (Figures 4D, E). The upregulated MTX-resistant genes intersected with the core gene module, finally obtaining 172 highly expressed genes in MTX-resistant osteosarcoma cells (Figure 4A). Considering the degradation and translational repression of target genes by miRNAs, the upregulated miRNAs were used to predict the regulated target genes and intersected with the downregulated MTX-resistant genes and the core gene module, and 49 target genes regulated by MTX-resistant miRNAs were finally identified (Figure 4A). They were combined to obtain 221 MTXDEGs for further analysis.

### 3.3 Construction and validation of a methotrexate resistance-related signature

**FIGURE 5:**
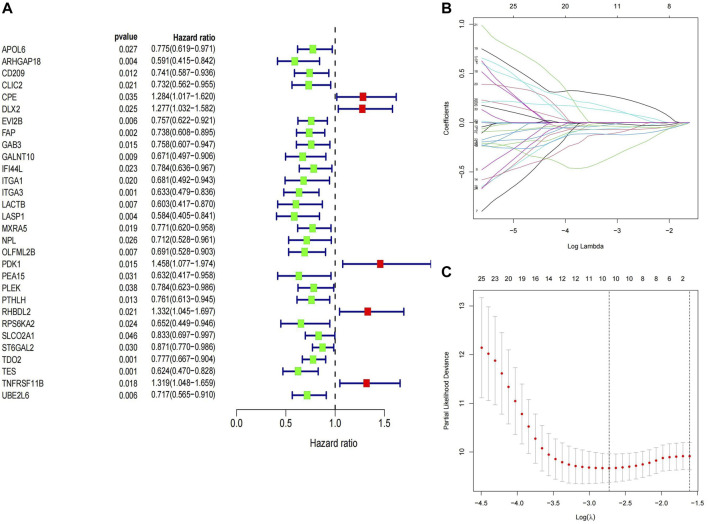
Construction of prognostic signature **(A)** Genes screened in univariate Cox regression **(B,C)** Best λ for LASSO regression.

We selected 82 samples with complete survival information from the TARGET-OS cohort for further analysis. To identify MTXDEGs significantly associated with prognosis, univariate Cox regression analysis was performed on 221 MTXDEGs, and 30 MTXDEGs were significantly associated with overall survival (*p* < 0.05) (Figure 5A). LASSO regression was then performed to screen genes for model building, and 10 genes were screened based on the best λ (Figures 5B, C). Based on the genes generated by LASSO regression, multivariate Cox regression identified 4 genes, *CPE*, *LASP1*, *PDK1*, and *LACTB*, as hub genes; Based on their expression levels, we developed a Riskscore signature of:t

$$\begin{array}{ll} Riskscore & =0.482850197357161*CPE-0.655999299291687*LASP1\\ & +0.44675499776076*PDK1-0.591342381951838*LACTB \end{array}$$ (22)

**FIGURE 6:**
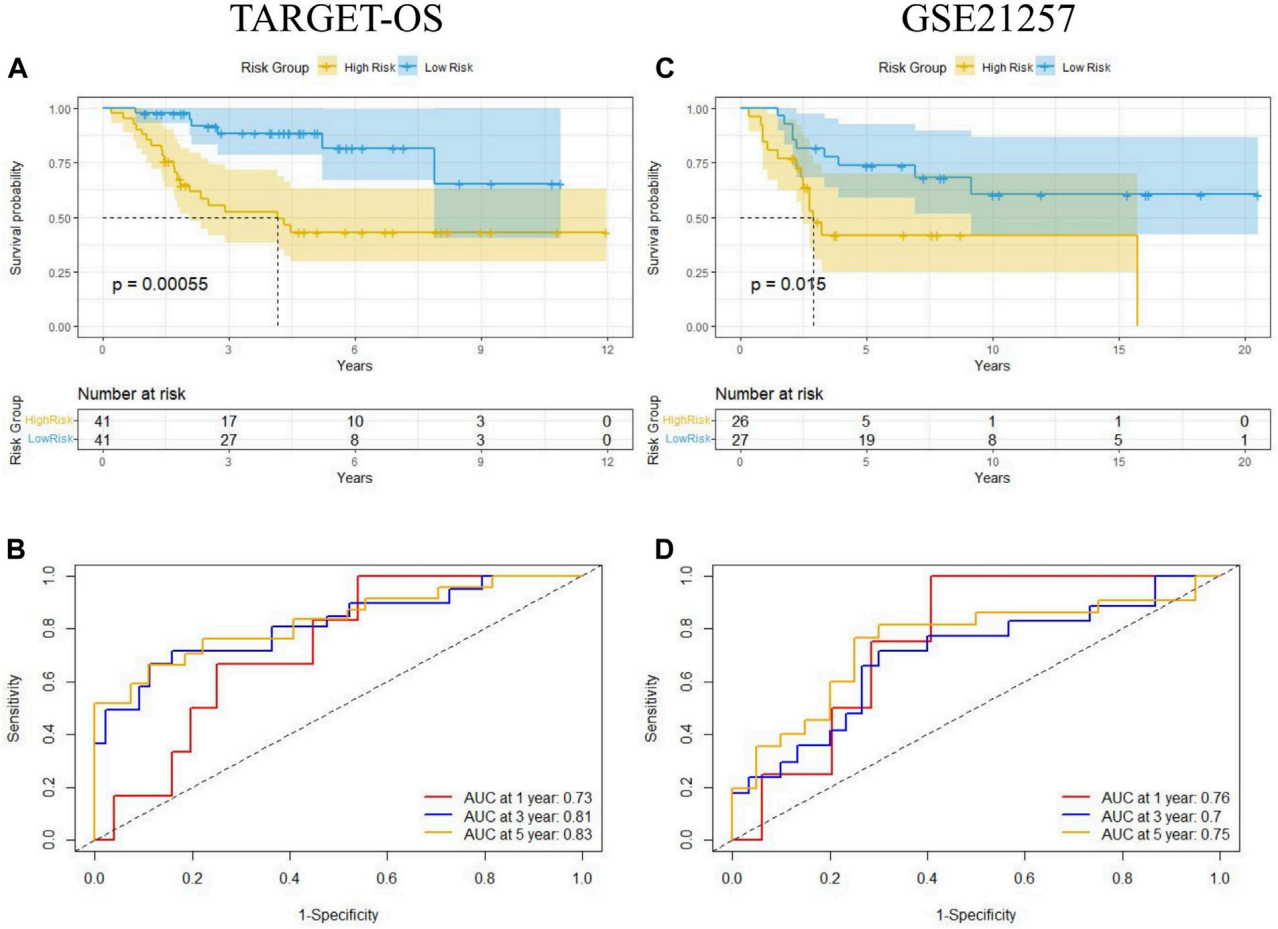
Kaplan-Meier analysis and time-dependent ROC analysis of MTX resistant signature in osteosarcoma. **(A,B)** Survival and ROC curves in training cohort (TARGET-OS). **(C,D)** Survival and ROC curves in validation cohort (GSE21257).

**FIGURE 7:**
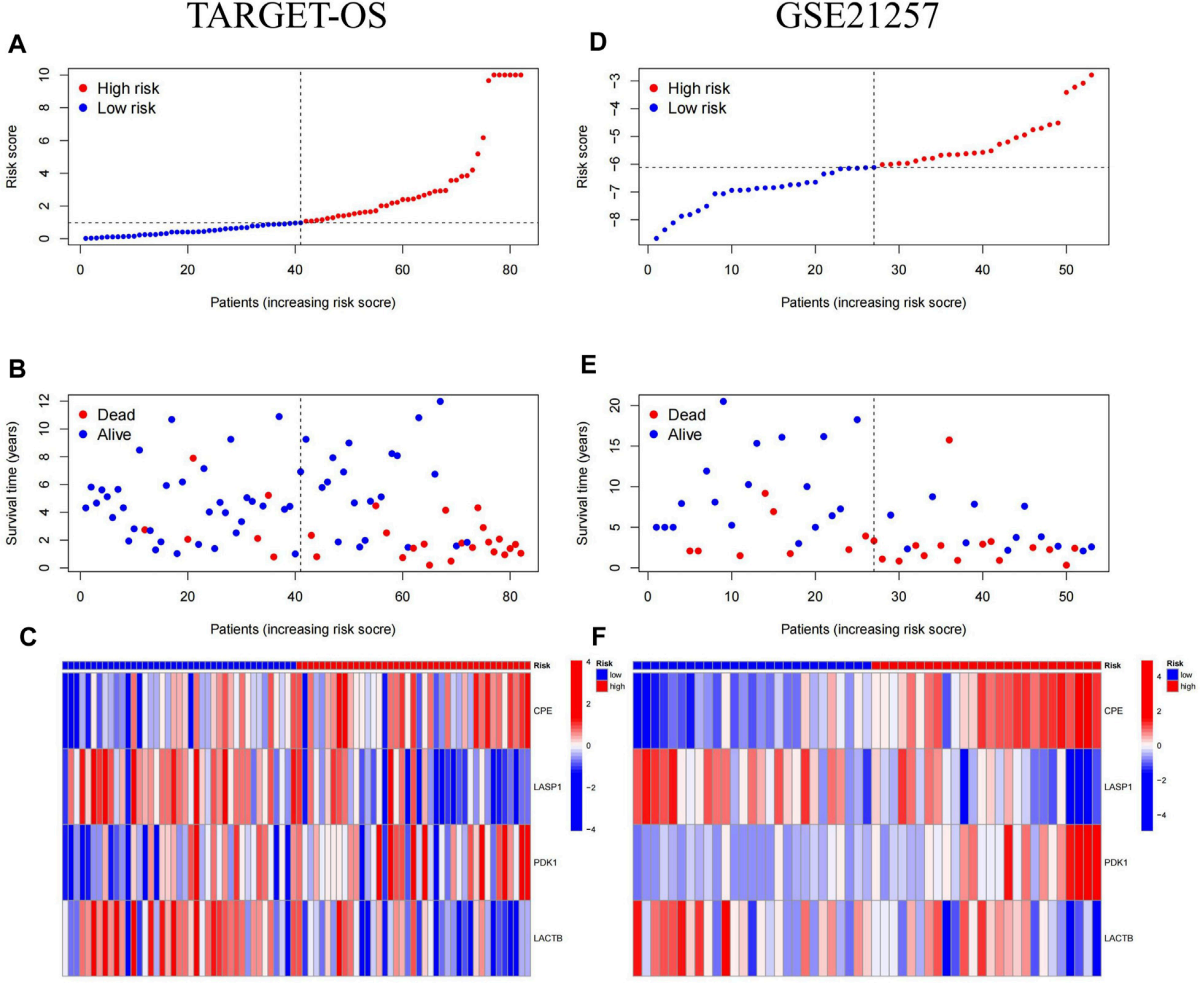
Risk score, survival status and hub genes expression heat map for MTX resistant signature in osteosarcoma. **(A–C)** Heatmap of Risk Scores, Survival Status, and Candidate Gene Expression in Training cohort (TARGET-OS). **(D–F)** Heatmap of Risk Scores, Survival Status, and Candidate Gene Expression in validation cohort (GSE21257).

The median of Riskscore was used as the threshold for the high-risk and low-risk groups. The Kaplan-Meier curve showed that the overall survival of the high-risk group was significantly lower than that of the low-risk group (*p* = 0.00055) (Figure 6A). The AUC of the 1-, 3-, and 5-year ROC curves were 0.73, 0.81, and 0.83, respectively (Figure 6B). Figure 7 shows the riskscore, survival status, and expression levels of the four candidate genes in the training cohort. The high-risk group had significantly higher risk scores and worse survival status than the low-risk group (Figures 7A–C). The above results show that the signatures can reasonably predict the overall survival of osteosarcoma patients in the training cohort. The validation cohort GSE21257 also classified patients into high-risk and low-risk categories using the median Riskscore. The Kaplan-Meier curves showed that the high-risk group had a shorter survival time compared to the low-risk group (*p* = 0.015) (Figure 6C), with AUC of 0.76, 0.7, and 0.75 for the 1-, 3-, and 5-year ROC curves (Figure 6D), respectively, which was consistent with the results of the training cohort. The riskscore and the expression of the 4 hub genes were also consistent with the training cohort (Figures 7D–F), indicating the prognostic value and reliability of the signature.

### 3.4 Construction of the nomogram based on MTXDEGs signature in TARGET-OS cohort

**FIGURE 8:**
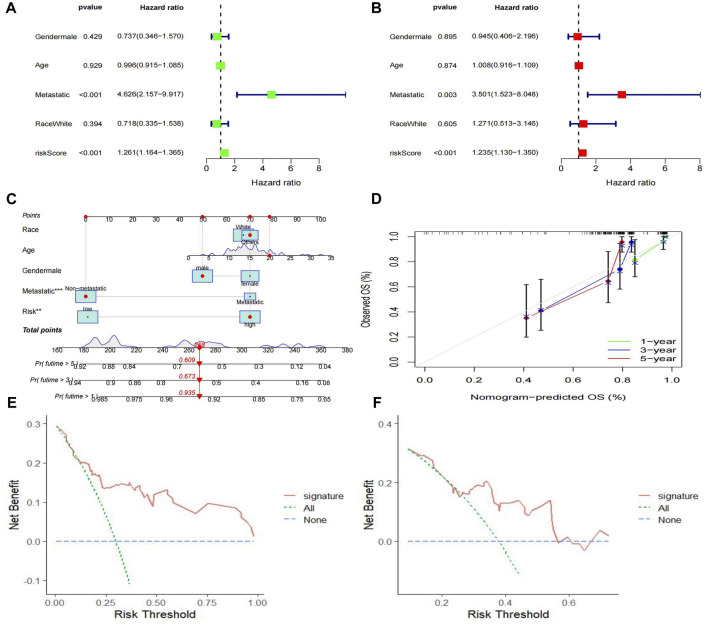
Construction of the nomogram predicting overall survival for osteosarcoma patients. **(A,B)** Forest plots for univariate and multivariate regression analysis. **(C)** A nomagram combines Riskscoresand clinical information. **(D)** Calibration curves for the accuracy of signature to predict 1,3,5-year survival. **(E–F)** Decision curve analysis for training **(E)** and validation **(F)** cohorts.

We used univariate and multivariate Cox regression analyses to verify whether the Riskscore generated by the 4 genes was an independent prognostic factor. The results showed that metastasis (*p* = 0.003) and Riskscore (*p* < 0.001) were independent prognostic factors for osteosarcoma (Figures 8A, B). We developed a nomogram using Riskscore and clinical data based on these significant factors (Figure 8C). The accuracy of the nomogram was evaluated using calibration curves at 1, 3, and 5 years (Figure 8D). The results showed that the calibration curves were very close to the ideal curve (a straight gray line with a slope of 1 through the origin of the coordinate axis). DCA curve was used to evaluate whether the model contributes to clinical treatment strategies. When the risk threshold probability varied between 0 and 1, the MTX-resistant gene signature achieved a higher net benefit in both the training and validation cohorts than the “treat all” and “treat none” strategies (Figures 8E, F). These results suggest that the MTX resistance gene signature performs well in clinical applications.

### 3.5 Functional analysis

**FIGURE 9:**
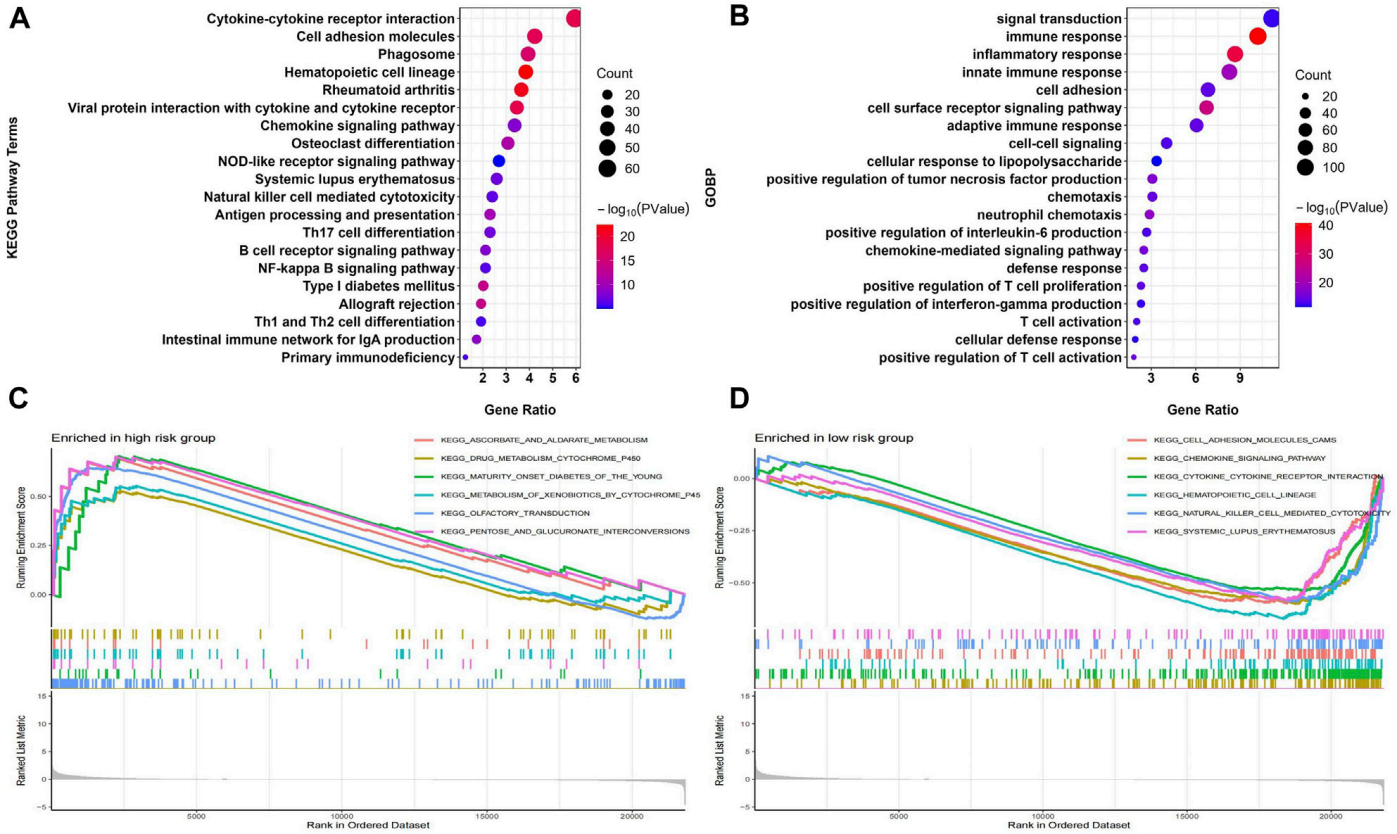
Construction of the nomogram predicting overall survival for osteosarcoma patients in the TARGET-OS cohort. **(A,B)** Forest plots for univariate and multivariate regression analysis. **(C)** A nomagram combines Riskscoresand clinical information. **(D)** Calibration curves for the accuracy of signature to predict 1,3,5-year survival.

A total of 1033 DEGs were identified between the high-risk and low-risk groups, which were analyzed for GO and KEGG enrichment. Figure 9 shows the top 20 most significantly enriched KEGG pathways and GO biological processes. KEGG analysis showed that DEGs were mainly enriched in Cytokine-cytokine receptor interaction, Cell adhesion molecules (CAMs), Phagosome, and other pathways related to tumor immune cell function and apoptosis (Figure 9A). GOBP analysis showed that DEGs were mainly enriched in signal transduction, immune response, inflammatory response, and other pathways related to immune function (Figure 9B). We also performed GSEA analysis to identify the underlying biological processes in the high-risk and low-risk groups. The results showed that ascorbate_and_aldarate_metabolism, drug_metabolism_cytochrome_p450, and other pathways related to metabolic function were enriched in the high-risk group (Figure 9C), and cell_adhesion_molecules_cams, chemokine_ signaling_pathway and other pathways related to immune function were enriched in the low-risk group (Figure 9D). The above results indicated that Riskscore is significantly associated with osteosarcoma immune status.

### 3.6 Immune cell infiltration

**FIGURE 10:**
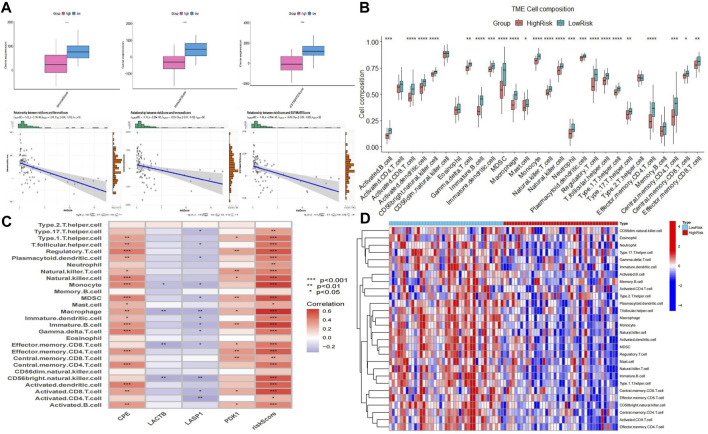
Analysis of immune infiltration between two groups. **(A)** Plot of differences between ImmuneScore, StromalScore, and ESTIMATEScore and correlation with Riskscore. **(B)** Bar plot of the difference between the two groups of 28 immune cells. **(C)** Correlation plot of four hub genes and Riskcore with immune cells. **(D)** Heatmap of immune cell infiltration between the two groups.

To study the differences in immune infiltration between the two groups of patients, we calculated the immune infiltration scores in the high- and low-risk groups using the ESTIMATE method. We noticed that the ImmuneScore, StromalScore, and ESTIMATEScore were significantly lower in the high-risk group than in the low-risk group. Riskscore and three immune scores were significantly negatively correlated (Figure 10A). We performed ssGSEA analysis and calculated the infiltration abundance of 28 immune cells. Surprisingly, the infiltration abundance of all 28 immune cells was lower in the high-risk group, with 23 immune cells being the most significant (Figure 10B). Among them, the hub genes *CPE* and *PDK1* were significantly positively correlated with 18 and 12 immune cells, and *LACTB* and *LASP1* were significantly negatively correlated with 4 and 13 immune cells. Riskscore was significantly positively correlated with 24 immune cells (Figure 10C). The heat map (Figure 10D) and the above results indicated that in the high-risk group, the infiltration of immune cells was significantly lower, and there were fewer immune cells in the tumor immune microenvironment, which may lead to a poor prognosis.

### 3.7 Drug sensitivity analysis

**FIGURE 11:**
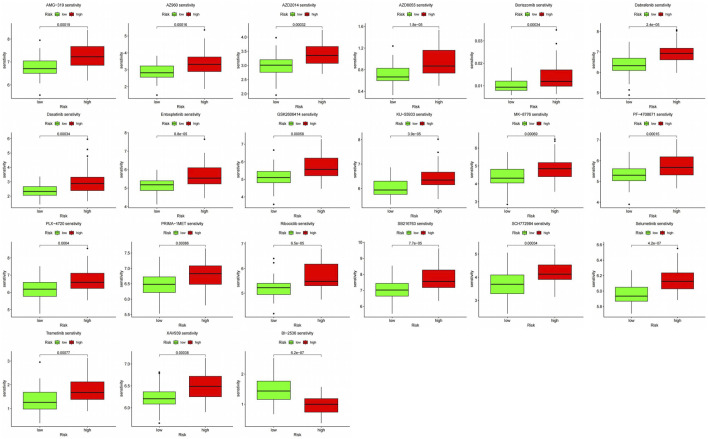
Response to chemotherapy and targeted therapy in two groups.

We further analyzed the response to chemotherapy and targeted therapy in the high-risk and low-risk groups. With a threshold *p* < 0.001, the results showed that the high-risk group showed higher resistance to 20 drugs, and the high-risk group was more sensitive to only one targeted drug, BI-2536 (Figure 11) [a small molecule inhibitor against PLK1 with a dual role in inducing apoptosis and impairing autophagy in neuroblastoma cells (Li et al., 2016)].

## 4 Discussion

### 4.1 Comparison of different multi-omics NMF methods

**FIGURE 12:**
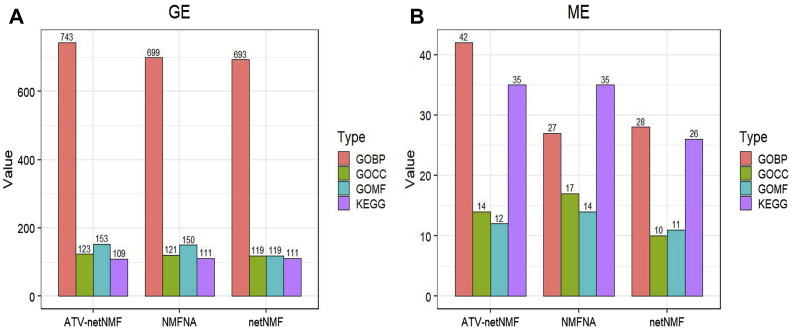
Comparison of ATV-netNMF with other methods. **(A,B)** Number of GO and KEGG pathways enriched by GE and ME modules.

ATV-netNMF successfully constructed co-expression networks between methylation and gene expression data, identified characteristic modules and characterized genes, and identified osteosarcoma biomarkers. To verify the performance of ATV-netNMF, we compared it with NMFNA and netNMF. We performed GO and KEGG enrichment analyses using the core modules identified by each of them, and the number of pathways and *p*-values of the pathways obtained are shown in Figures 12A, B and Table 1. It can be seen that the ME and GE core modules identified by ATV-netNMF enriched more pathways, and the *p*-values of the significant pathways were lower compared to the other methods, which suggests that the modules identified by ATV-netNMF may contain more biological information related to osteosarcoma, and enriched to more significant pathways. This is because the basis vectors obtained from ATV-netNMF decomposition are more sparse than netNMF and NMFNA, eliminating some noise in the data and enhancing some features and details.

**TABLE 1:** KEGG pathways that appear in three methods in the GE module.

KEGG pathway	ATV-netNMF	NMFNA	netNMF
	*p*-value	*p*-value	*p*-value
Cytokine-cytokine receptor interaction	2.00E-21	**8.06E-22**	5.61E-14
Osteoclast differentiation	3.33E-20	**1.98E-20**	3.71E-18
Cell adhesion molecules	**6.05E-16**	1.43E-15	1.35E-15
Rap1 signaling pathway	**4.33E-11**	1.61E-10	4.25E-10
NF-kappa B signaling pathway	**4.58E-08**	1.64E-07	3.86E-05
Th1 and Th2 cell differentiation	**2.07E-07**	2.15E-07	1.70E-07
Pathways in cancer	6.19E-07	**3.73E-07**	3.26E-06
JAK-STAT signaling pathway	**6.83E-07**	2.14E-06	2.04E-06

The bold value indicates the Minimum values.

To further evaluate the applicability of ATV-netNMF and the contribution between individual modules, we downloaded gene expression data and miRNA data from cancer samples in the High-Risk Wilms Tumor (TARGET-WT), Breast Invasive Carcinoma (TCGA-BRCA), and Lung Adenocarcinoma (TCGA-LUAD) datasets from the UCSC Xena database (https://xenabrowser.net/datapages/) for analysis. Specifically, we calculated the Pearson correlation coefficients between the reconstructed matrices $R_{11}^{\prime },R_{12}^{\prime }\text{ and}R_{22}^{\prime }$ and the original matrices $R_{11},R_{12}\text{ and}R_{22}$ in netNMF, NMFNA, netNMF + ATV, and ATV-netNMF, respectively. As can be seen from Table 2, the reconstruction of both R11 and R22 outperforms the netNMF method for all four datasets under the separate constraints of graph regularization and adaptive total variation. The proposed ATV-netNMF method also outperforms the previous methods in reconstructing all four datasets. The above results confirm that graph regularization and adaptive full mutation help improve the reconstruction performance of the methods, and the proposed ATV-netNMF can be used to detect co-expression modules from multiple diseases and genetic data.

**TABLE 2:** Pearson’s correlation coefficients between the three original matrices and the three reconstructed matrices obtained by different algorithms in different data sets.

		netNMF	NMFNA	netNMF + ATV	ATV-netNMF
TARGET-OS	$\text{corr}\left(R_{11},R_{11}^{\prime }\right)$	0.8737	0.8768	0.8792	**0.8813**
	$\text{corr}\left(R_{12},R_{12}^{\prime }\right)$	0.9038	**0.9060**	0.8958	0.9001
	$\text{corr}\left(R_{22},R_{22}^{\prime }\right)$	0.9170	0.9224	0.9194	**0.9233**
TARGET-WT	$\text{corr}\left(R_{11},R_{11}^{\prime }\right)$	0.8678	0.8682	0.8694	**0.8762**
	$\text{corr}\left(R_{12},R_{12}^{\prime }\right)$	**0.9237**	0.9225	0.9166	0.9193
	$\text{corr}\left(R_{22},R_{22}^{\prime }\right)$	0.9167	0.9163	0.9200	**0.9204**
TCGA-BRCA	$\text{corr}\left(R_{11},R_{11}^{\prime }\right)$	0.9285	0.9293	0.9291	**0.9296**
	$\text{corr}\left(R_{12},R_{12}^{\prime }\right)$	0.9190	**0.9287**	0.9213	0.9249
	$\text{corr}\left(R_{22},R_{22}^{\prime }\right)$	0.7579	0.7627	**0.7764**	0.7708
TCGA-LUAD	$\text{corr}\left(R_{11},R_{11}^{\prime }\right)$	0.9094	0.9052	0.9133	**0.9141**
	$\text{corr}\left(R_{12},R_{12}^{\prime }\right)$	0.8943	0.9145	0.9162	**0.9165**
	$\text{corr}\left(R_{22},R_{22}^{\prime }\right)$	0.7894	0.7902	0.7892	**0.7930**

The bold value indicates the Maximum values.

### 4.2 Biological functional analysis

Patients with localized osteosarcoma can be cured with neoadjuvant chemotherapy and surgical resection in up to 70% of cases, but survival rates for chemotherapy-resistant and metastatic patients are less than 20%. There is a significant correlation between response to chemotherapy and the prognosis of osteosarcoma, and one of the main challenges is inherent or acquired resistance. Methotrexate is used as a common strategy for chemotherapy in osteosarcoma, and patients with MTX resistance often experience tumor recurrence and metastasis. Therefore, the discovery of reliable biomarkers and the search for new therapeutic targets are essential to improve the clinical prognosis of osteosarcoma. To provide new prognostic predictors and immunotherapies for chemotherapy-resistant patients with osteosarcoma, our study identified meaningful predictive biomarkers by comprehensively analyzing multi-omics genetic data of osteosarcoma.

In this study, first, ME, GE, and ME-GE networks were constructed based on two genetic data. Then, these networks were decomposed under the constraints of total adaptive variance and graph regularization, while co-expression modules were efficiently identified, which is the highlight of ATV-netNMF. Finally, the core networks were analyzed for GO and KEGG enrichment. Compared with NMFNA and netNMF, ATV-netNMF could identify more osteosarcoma-related GO terms and pathways, indicating that ATV-netNMF could effectively detect modules and characterize genes. Based on that, combined with MTX-resistant mRNA and miRNA data, we established a new 4-gene prognostic signature for osteosarcoma, including two high-risk MTXDEGs (CPE, PDK1) and two low-risk MTXDEGs (LASP1, LACTB). We can categorize patients into high-risk and low-risk subgroups based on the risk scores derived from this predictive model. *CPE* is highly expressed in MTX-resistant cells. *PDK1*, *LASP1*, and *LACTB* were lowly expressed in MTX-resistant cells and were target genes regulated by MTX-resistant miRNAs. *CPE* is a prohormone processing enzyme that is usually overexpressed in osteosarcoma cell lines (Shi et al., 2020), and the downregulation of *CPE* inhibits the migration and invasive ability of osteosarcoma cells. Overexpression of a splice variant of *CPE*, CPE−∆N , promotes the growth and metastasis of osteosarcoma cells (Li et al., 2016). *PDK1* is a key rate-limiting enzyme of the tricarboxylic acid cycle. *PDK1* expression was suppressed in DXR-resistant osteosarcoma cells (Zhang et al., 2021), consistent with our experimental findings that *PDK1* is downregulated in MTX-resistant osteosarcoma. *PDK1* was overexpressed in osteosarcoma, multiple myeloma, acute myelogenous leukemia, and breast cancer (Zhang et al., 2020). *LASP1* is an actin-binding protein, and overexpression of *LASP1* is associated with poor prognosis in patients with gastric cancer (Keckesova et al., 2017). After the downregulation of *LASP1*, the resistance of osteosarcoma cells to cisplatin was reduced, the IC50 decreased, and the knockdown of *LASP1* could result in the inhibition of the proliferation of osteosarcoma cells (Chang et al., 2022). *LACTB* is a mitochondrial protein that is highly expressed in skeletal muscle, heart, and liver (Keckesova et al., 2017). In breast and colorectal cancers, low expression of *LACTB* predicts a poorer prognosis for patients (Zhang et al., 2018; Li et al., 2019). However, in glioblastoma, *LACTB* overexpression inhibits cancer cell proliferation (Hu et al., 2022).

Based on these four genetic features in the risk model, we performed a comprehensive analysis and assessment of the two subgroups, which showed a significant difference in survival time between patients in the high-risk and low-risk groups. In addition, our KEGG and GO enrichment analyses showed that many immune and tumor-related pathways were enriched. We further performed GSEA analysis and found that metabolism-related pathways such as ascorbate and aldate metabolism, cytochrome P450 in drug metabolism to xenobiotics, adolescent diabetes mellitus, glucose metabolism, etc., were enriched in the high-risk group. In the low-risk group, pathways related to immune function, such as cell adhesion molecules, hematopoietic factor signaling pathways, cytokine-receptor interactions, natural killer cell-mediated cytotoxicity, and hematopoietic cell lines, were enriched.

We further analyzed the infiltration status of various immune cells using ESTIMATE and ssGSEA methods to investigate the immune infiltration differences between the two subgroups. The findings showed that the immune, stromal, and estimate scores were significantly lower in the high-risk group than in the low-risk group. A significant negative correlation existed between our calculated risk scores and the three immune scores. In addition, the infiltration abundance of all 28 immune cells was lower in the high-risk group, with significant differences in the infiltration abundance of 23 immune cells. Riskscore showed a significant positive correlation with 24 immune cells. Osteosarcoma is considered a “cold tumor” in terms of immunogenicity. In the high-risk group, the infiltration of immune cells was significantly lower. The lower number of immune cells in the tumor immune microenvironment may lead to a worse prognosis. The prognosis of high-risk patients may be improved by increasing the immune reactivity.

The need for high iron is an important feature of many cancer cells (Torti and Torti, 2013), and many cancer cells also have higher basal levels of intracellular unstable iron compared to normal cells. In osteosarcoma cell lines, higher levels of iron in the cells enhanced ascorbate-induced pharmacological toxicity. They made the cells more sensitive to ascorbic acid, thereby increasing the resistance of MNNG/HOS and U2OS cells to ascorbate-induced drug toxicity (Schoenfeld et al., 2017; Zhou et al., 2020). P450 is a major phase I drug-metabolizing enzyme that activates a variety of potent chemical carcinogens. Previous studies have confirmed that resistance to chemotherapy in osteosarcoma is associated with cytochrome P450, and our results may provide evidence for these previous findings (Ferrari et al., 2009). The researchers found that low expression of monocytes in patients with osteosarcoma reduced the expression of cell adhesion molecules and chemokine receptors, and they also exhibited decreased chemotactic function, i.e., the ability of monocytes to enter the tumor site and initiate an anti-tumor immune response (Mason, 1970). Our findings support the notion that patients with higher monocyte expression have monocytes that can migrate to areas of inflammation that respond to chemotactic proteins, thereby improving survival (Tuohy et al., 2016). Lower expression of regulatory T cells predicts shorter overall survival (Biller et al., 2010); However, a higher degree of T cell infiltration predicts increased survival (Scott et al., 2018). In summary, there is a cross-talk between immune-metabolic responses and tumor-related pathways that lead to tumorigenesis and chemoresistance. The above evidence suggests that immunomodulation has beneficial effects on prognosis. Immune dysfunction promotes tumor progression and drug resistance; therapeutic strategies to reverse immune dysfunction can improve patient prognosis, and identifying relevant biomarkers would further improve clinical response.

Survival of patients in the high-risk and low-risk groups was significantly correlated with their sensitivity to chemotherapy, and changes in therapeutic strategies are necessary to improve outcomes in patients who are insensitive to chemotherapeutic agents. Therefore, we analyzed the sensitivity of patients in both groups to commonly used chemotherapeutic and targeted drugs. The results showed that the high-risk group resisted most drugs, and BI-2536 may be considered a therapeutic candidate for the high-risk group.

This study has some limitations. Although this study is based on multiple datasets and multi-omics data, further experimental validation still needs to be improved. In subsequent studies, we need to conduct more experiments to clarify the underlying molecular mechanisms of MTXDEGs.

## 5 Conclusion

We proposed an adaptive total variant constrained-based netNMF multi-omics analysis method that integrates and efficiently identifies co-expression modules and characteristic genes in osteosarcoma methylation and gene expression data. Combined with the methotrexate-resistant multi-omics data, we identified a four-gene-based prognostic model with predictive solid ability for patient survival, immune microenvironment, and immunotherapeutic efficacy, which provides direction for new therapeutic strategies. In conclusion, the MTX resistance-associated model based on ATV-netNMF offers new targets for researchers to explore the mechanism of action of chemoresistance in osteosarcoma.

## Data Availability

The original contributions presented in the study are included in the article/Supplementary Material, further inquiries can be directed to the corresponding authors.
